# Dangerous Properties of Dusts

**Published:** 1882-06

**Authors:** 


### POPULAR SCIENCE DEPARTMENT.

### DANGEROUS PROPERTIES OF DUSTS.

Professor Abel, F.R.S., lately delivered a lecture at the Royal Institution on "Some of the Dangerous Properties of Dusts," of which a short abstract is here given :

The liability to the development of fire or of fire sufficient to char or inflame portions of flour by the stoppage of the feed of grain, appears from all accounts to be extremely difficult to guard against, and to have been the cause of many serious calamities ever since the Tradeston explosion, examples of which are the great explosion of six mills at Minnesota in 1878, when eighteen lives were lost and much property was destroyed ; and the fatal and destructive explosion of a flour mill at Macclesfield in September last, which has been made the subject of a report to the Home Office by Mr. Richards, of the Board of Trade. It appears to be the opinion of experienced men in the trade that although special attention to the feed arrangements may reduce the number of explosions, this cause of accidents is almost impossible to guard against; while, on the other hand, many fires or explosions ascribed to it have been due to the employment of naked lights in mills near localities where the air is laden with flour dust. Considering that flour and rice mill owners have to bear the burden of very heavy rates of insurance, it is to their interest, independently of their responsibilities as the guardians of the lives of their workmen, to adopt most stringent regulations and efficient precautionary measures for abolishing this source of danger, and to devote their energies to the application of improved arrangements for reducing the quantity of dust which passes away from the millstones, and from other parts of a flour mill.

The important part played by coal-dust, which exists in greater or less abundance in all coal mines, in aggravating and extending the injurious effects of fire-damp explosions, was originally pointed out early in 1845 by Faraday and Lyell, when they reported to the Home Secretary the result of their inquiry into an explosion which occurred at Haswell Collieries in September, 1844.

Ten years after the publication of Faraday and Lyell's report, M. De Souich, an eminent French mining engineer, published as original some very similar observations made by him on examining the effects of a coal mine explosion at Firminy; he noticed, moreover, that men near the pit's mouth had received burns, while others who were in the workings near the seat of the explosion, but out of the main air current, escaped unhurt, and he ascribed this to the action of coal-dust in carrying flame along the principal air-way. Later on, De Souich extended his inquiries into the part played by coal-dust in explosion, and the subject was afterward pursued, from time to time, in France, by Verpilleux and other authorities in mining engineering, and especially by M. Vital, in 1875, when an explosion occurred at Campagnac, the destructive effects of which appeared to him in a great measure ascribable to coal-dust. Vital made experiments upon a very small scale, for the purpose of ascertaining whether flame, such as that projected into the air of a mine by the firing of a charge of powder, in a very strong blast hole, was increased in size by the presence of suspended coal-dust; and soon afterward Mr. W. Galloway commenced a series of experiments of similar nature, but upon a larger scale, which he has continued from time to time up to the present date; while Messrs. Marreco and Morison, in connection with the

North of England Institute of Mining Engineers, and a committee of the Chesterfield and Derby Institute of Engineers, have also contributed valuable experimental data bearing upon the influence exerted by coal-dust, not merely in increasing the magnitude of explosions resulting from the ignition of mixtures of fire-damp and air, but also in propagating or even actually developing explosions, when only small quantities of fire-damp are present in the air of a mine, or where fire-damp is believed to be entirely absent. The conclusion to which Mr. Galloway was led by his earlier experiments was to the effect that coal-dust, when thickly suspended in air, had not the power to originate an explosion, or to carry on to any distance the flame from a blown-out shot, but that the presence in the air of such small quantities of fire-damp (2 per cent and under) as an experienced miner would fail to detect by means of his Davy lamp, with which the gas is generally searched for, would impart to a mixture of coal-dust and the air the property of burning and carrying flame. But he held the view, at the same time, that a fire-damp explosion in one part of a mine might be propagated to some extent by coal-dust raised by the effects of the explosion in parts of the mine where no fire-damp existed. Marreco, on the other hand, considered that the results of certain experiments made in the entire absence of coal-dust, by firing shots in air, travelling at some considerable velocity, and containing coal-dust thickly suspended in it, warranted the conclusion that coal-dust also might, under certain conditions, originate an explosion as well as carry it on to some considerable extent. The results obtained by the corresponding experiments of the Chesterfield Committee appear to support this view, and Mr. Galloway has also, by his later experimental results, been led to the same conclusion, and considers that the results of his examination into the effects produced by some of the most serious of recent coal-mine explosions (at Penygraig, Risca, and Seaham) demonstrate that those explosions were chiefly, if not entirely, attributable to coal-dust.

The strong impression entertained by many, during the inquiry into the great explosion at Seaham Collieries, in September, 1880, that coal-dust might have had much to do with the accident, and that the explosion was possibly even entirely due to the ignition of coal-dust by blown-out shot in the absence of any fire-damp, led to Mr. Abel being requested by the Home Secretary to make experiments with samples of dust collected from collieries in different parts of the kingdom where explosions had occurred.

The results of experiments conducted with great care and on an extensive scale at a colliery in Lancashire, where a constant supply of fire-damp was brought to the pit's mouth from a so-called blower, confirmed the fact demonstrated by M. Vital and Mr. Galloway, that the propagation of fire by coal-dust, when thickly suspended in air, is established or greatly promoted by the existence, in the air, of a proportion of fire-damp, which may be so small as to escape detection by the means ordinarily employed (such, for example, as exists in the return air of a well ventilated mine).

It was also established that a mixture of fire-damp and air approaching in proportions those required to be explosive, would be ignited by a flame if only a small proportion of dust were floating in it. Further, it was demonstrated that, although those dusts which were richest in inflammable matter, and most finely divided, were the most prone to inflame and to carry flame, in the presence of small quantities of fire-damp, some dusts which contain coal only in comparatively small proportions were as sensitive as other much richer in inflammable matter; and that even perfectly non-combustible dusts possessed the property of establishing the ignition of air and gas mixtures which, in the absence of dust, were not ignited by a naked flame. The action of non-combustible dusts appeared to be due to physical peculiarities of the finely divided matter, and to be perhaps analogous to the contact action so well known to be possessed by platinum and some other bodies, whereby these bring about the rapid oxidation of gases which, in their absence, may exist intact in admixture with oxygen or air.

Although it may be very doubtful whether coal-dust, in the complete absence of fire-damp, can be connected with the production of extensive explosions, as has been recently maintained by some, there can be no question that, in the presence of only very small quantities of fire-damp, it may establish and propagate violent explosions; and that, in the case of a fire-damp explosion, the dust not only, in most instances, greatly aggravates the burning action, and increases the amount of after damp, but that it may also, by being raised and swept along by the blast of an explosion, carry the fire into workings where no fire-damp exists, and thus add considerably to the magnitude of the disaster. The supposition that extensive coal-mine explosions may be produced by coal-dust alone, in the complete absence of gas, necessitates the fulfillment of conditions which cannot be at any rate very exceptional, but its acceptance is unnecessary to add to the formidable character of coal-dust as an agent of destruction in mines. The possibility of dealing with the dangerous dust in mines should, therefore, be as much an object of earnest work as has been the improvement of ventilating arrangements in mines.

The actual removal of dust accumulations being in most instances impracticable, the laying of the dust by an efficient system of watering the mine ways is a matter deserving serious attention. Although in some instances such a measure is not readily applicable without injury to the workings, it has been already proved in some districts to be unobjectionable and susceptible of very beneficial application. The employment of deliquescent substances (calcium chloride, sea salt, etc.) in conjunction with watering, has also been advocated and tried to some extent with success.

